# Weighing costs and benefits of mating in bushcrickets (Insecta: Orthoptera: Tettigoniidae), with an emphasis on nuptial gifts, protandry and mate density

**DOI:** 10.1186/1742-9994-9-19

**Published:** 2012-08-15

**Authors:** Gerlind U C Lehmann

**Affiliations:** 1 Department of Biology, Behavioural Physiology, Humboldt-Universität zu Berlin, Invalidenstrasse 43, Berlin 10115, Germany

**Keywords:** Tettigoniidae, Bushcrickets, Katydids, Sexual selection, Female choice, Economics of mating, Different allocation hypothesis, Mating decisions, Spermatophores, Acoustic communication

## Abstract

Sexual selection is a major force driving evolution and is intertwined with ecological factors. Differential allocation of limited resources has a central role in the cost of reproduction. In this paper, I review the costs and benefits of mating in tettigoniids, focussing on nuptial gifts, their trade-off with male calling songs, protandry and how mate density influences mate choice. Tettigoniids have been widely used as model systems for studies of mating costs and benefits; they can provide useful general insights. The production and exchange of large nuptial gifts by males for mating is an important reproductive strategy in tettigoniids. As predicted by sexual selection theory spermatophylax size is condition dependent and is constrained by the need to invest in calling to attract mates also. Under some circumstances, females benefit directly from the nuptial gifts by an increase in reproductive output. However, compounds in the nuptial gift can also benefit the male by prolonging the period before the female remates. There is also a trade-off between adult male maturation and mating success. Where males mature before females (protandry) the level of protandry varies in the direction predicted by sperm competition theory; namely, early male maturation is correlated with a high level of first inseminations being reproductively successful. Lastly, mate density in bushcrickets is an important environmental factor influencing the behavioural decisions of individuals. Where mates are abundant, individuals are more choosey of mates; when they are scarce, individuals are less choosey. This review reinforces the view that tettigoniids provide excellent models to test and understand the economics of matings in both sexes.

## Introduction

Sexual selection is a major force driving evolution, based on variation in reproductive success among individuals of differing phenotypes [1]. Males and females often differ profoundly in selected traits and sex differences in mating competition are a notable feature, usually attributed to differences in parental investment. There are competing demands on a parent between how much it invests improving their current offspring’s chance of survival (and hence reproduction) and how much it should invest in additional matings to create more offspring [2]. The investment pattern biases the ratio of sexually receptive females to males (the operational sex ratio, [3], generating intense competition between members of the more abundant mate-ready sex, usually males [4-6]. This creates opportunities of members of both sexes to adjust their mating decisions and investment in response to the decisions of other members of the guild, the game theoretic approach [7-9]. Sexual selection is also influenced by ecological factors [1,10,11], with environmental-dependent heterogeneity inducing spatial and temporal variation in sexual selection. Therefore including ecological conditions into the research about sexual selection might be able to resolve conflicting results obtained from studies of sexual traits [12]. Selection is a complex process involving many life history choices, such as the quantity of energy reserves to allocate to reproduction [13]. Where the reproductive capacity of an investing individual relies on internal energy reserves, the reproductive effort is limited by the amount of their reserves. Under such restricted conditions, individuals have to allocate resources to somatic or reproductive functions. These trade-offs have been described by the “Y” model of resource allocation [14]. The core idea of this model is that the differential allocation of limited internal resources has a central role in the cost of reproduction and other life-history trade-offs [15-17]. Theory shows that mating ‘economy’, i.e. the costs and benefits that mediate male–female interactions, is crucial for the extent to which traits are under sexual selection [18,19]. However, the economy of sexual traits has been assessed [20], including costs and benefits of producing and expressing traits, and costs and benefits of these traits for the opposite sex [21] in surprisingly few systems.

## Bushcrickets as model systems

**Figure 1:**
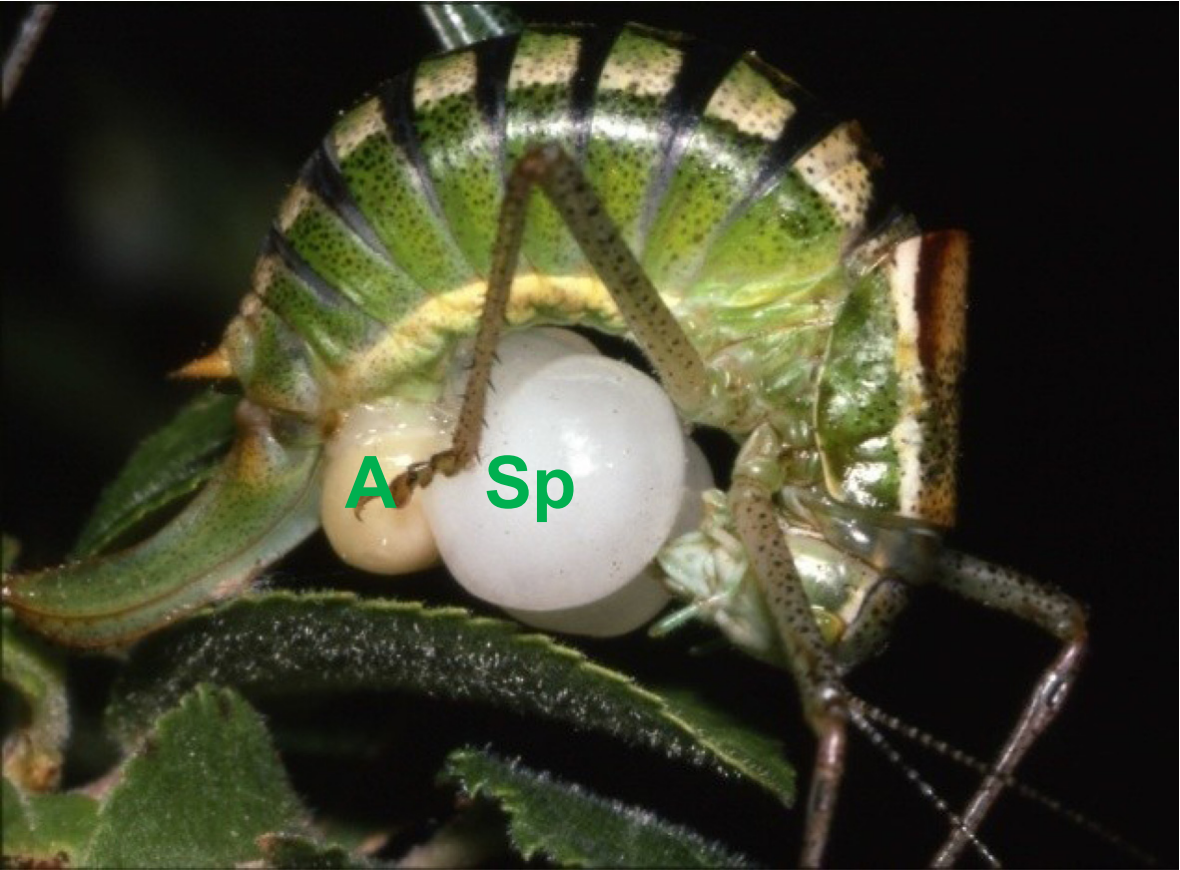
**A freshly mated bushcricket female (*Poecilimon thessalicus*) bends her head between her forelegs to consume the male provided spermatophylax (Sp).** The sperm starts to enter from the ampulla (**A**), placed near to the female genitalia.

Two aspects make bushcricket (Orthoptera: Tettigoniidae) species appropriate study organisms for investigations of the costs and benefits of mating. Firstly, tettigoniids attracted early attention because the male’s produce large nuptial gifts [22-24], transferring a food gift to a female in exchange for mating [25,26]. The nuptial gift, or spermatophylax, is a large, gelatinous offering, attached to the ampulla, which contains the ejaculate and sperm. Together the spermatophylax and the ampulla are called the spermatophore (Figure1). Crucial for the understanding of the function and evolution of spermatophores is sperm competition, with the spermatophylax having a role in protecting the ampulla, and therefore increasing the quantity of sperm transferred [25-27].

**Figure 2:**
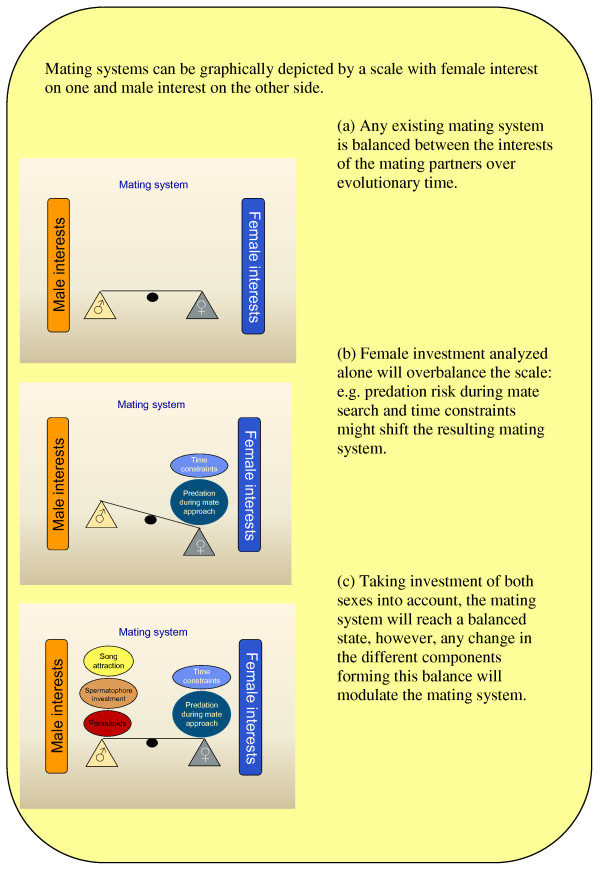
**Weighing costs and benefits in the mating system of a model tettigoniid species.** Mating systems can be graphically depicted by a scale with female interest on one and male interest on the other side.

Secondly, tettigoniids are model organisms for studies on sexual selection because of their use of acoustic signalling for mate attraction and intrasexual competition [26,28-31]. These pointed early observers towards the function that acoustic signals can play in mating. Acoustic signals can be analyzed in detail, their signals can be manipulation and they can be presented to receivers independent of the actual sender [28,29]. Analyzing the costs and benefits of mating for both sexes provides general insights into factors shaping mating systems. Understanding of the evolution and maintenance of mating systems is enhanced when a range of factors is considered, such as nutritional ecology [32] and animal decision-making [33]. I review some of the costs and benefits associated with reproduction in both male and female bushcrickets (Figure2). Particular attention is paid to two aspects of male reproductive economy (nuptial gifts and acoustic signalling), the costs and benefits of nuptial feeding for females, and the occurrence of protandry in combination with sperm precedence and mate density as an environmental factor influencing mate choice. These factors have been poorly covered by previous reviews of mate choice.

## Costs of nuptial gift provisioning for male bushcrickets

Males from a variety of insect orders provide their mates with a nutrient gift during or prior to copulation [34]. Male feeding during copulation is widespread in Orthoptera, and has evolved independently several times [35]. In tettigoniids, males transfer a nutritious spermatophylax attached to the sperm-containing ampulla [22-25]. The spermatophylax and ampulla constitute the spermatophore, which can be extremely large, weighing up to 30% of the male’s body mass [36-39]. After the spermatophore is attached to the female, she feeds on the spermatophylax (Figure1), while the sperm and ejaculate are transferred from the ampulla into her spermatheca. The female then consumes the ampulla. The spermatophylax protects the sperm by preventing the ampulla from being removed by the female [25]. The spermatophylax size should be large enough to ensure complete transfer of the ejaculate, with sub-optimal sized spermatophylaces resulting in impaired insemination [27]. The spermatophylax can, therefore, be considered a male mating effort, originally serving an ejaculate-protection function [26,34]. These gifts confer considerable benefits to females (see next section), and females generally prefer heavier males, who provide larger spermatophylax meals [40-42]. While larger nuptial gifts may provide direct nutritional benefits to females, they may also be viewed as a means by which males can overcome the resistance of the female to accepting larger ejaculates by lengthening the time before the female removes the sperm containing ampulla [43,44]. Substances in the ejaculate manipulate female mating behaviour in a dose dependent manner. The more sperm and ejaculate that are transferred the longer the female remains unreceptive to further mating [25,26]. Sexual selection should therefore strongly act on male bushcrickets to maintain or increase spermatophore investment. However, substantial costs incurred by males can limit such investment.

Previously, bushcricket mating systems were considered as a model for cooperation between the sexes, with males protecting their ejaculates and females receiving a nutritious offering [26]. Parker [45], however, pointed out the potential for conflict of interest, with the two sexes having different optima for spermatophore size, composition and quality. The size and quality of the spermatophylax may be the result of antagonistic coevolution between the sexes [43,44]. Testing this hypothesis requires study of the costs and benefits to both sexes of spermatophores (and their constituent parts) of varying sizes [46].

Tettigoniid males show considerable variation in spermatophore investment among species [36-39]. Comparative analyses, while sometimes lacking the power of experimental studies, nonetheless do indicate selection for a large spermatophylax might be a driving force for large male body mass. Spermatophore mass scales with male body mass for a large number of species [36,39,47]. The mass of both the spermatophylax and ampulla predictably scale with male body mass across bushcricket species [36,38,48,49], including the genus *Poecilimon*[39]. Not surprisingly, sperm number [38,39,48] and testis weight [50] shows the same positive relationship with male body mass in comparative studies. Spermatophylaces should be at least large enough to enable the transfer of the majority of sperm into the female [25,26]. One implication, supported in the literature, is that spermatophylax and ampulla mass should co-vary [36-38,48,49], as should sperm number and spermatophylax size [38,48]. However, these correlations are only moderate across 31 *Poecilimon* species [39], and appear to be influenced by the acoustic communication system, with species having a bidirectional acoustics system investing less than species having an unidirectional acoustic communication system [51].

In tettigoniids, variation in spermatophore investment between populations has been poorly unstudied. A review of spermatophore size in the genus *Poecilimon* examined intraspecific variation across several populations from a number of species, showing large differences in the total and relative spermatophore size [39]. *Poecilimon veluchianus* populations differ remarkably in body size [52]. These differences can be heritable, with some traits showing genetic linkage to the female X-chromosome [53]. *P. veluchianus* may in fact consist of two sub-species, since there are stable body size differences and a small but notable hybrid disadvantage in the F_1_ generation between some populations [54]. Spermatophore production is largely determined by body mass [54,55]. With this body size difference in mind, it is not surprising that males from both subspecies differ largely in their spermatophore investment pattern. Males from the smaller subspecies *P. v. minor* produce smaller spermatophores and remate more quickly in the field than the larger and heavier males of *P. v. veluchianus*[54]. Large body size differences have been also found between populations of the related species *P. thessalicus* as well [56]. This has been interpreted as an adaptation to differences in food availability caused by differences in precipitation between habitats. Absolute differences in population mean body size are coupled with changes in the sexual size differences: populations; with populations of larger overall mean body size having a reduced male body size disadvantage compared to populations with smaller mean body size [56]. Whether these body size differences result in different patterns of spermatophore investment and mating strategies remains to be investigated.

## Strategic allocation of spermatophores in male bushcrickets

Spermatophore provisioning, including nutrients and/or manipulative chemicals, by individual males might be under strong sexual selection. Models of ejaculate expenditure [57] and nuptial gift allocation [58] assume that there will be a trade-off in males between resources spent on current and future reproduction. Theory predicts that males should strategically allocate sperm and ejaculates with regard to their condition at the time of mating [57,59-61]. Tettigoniid males of the species *Poecilimon zimmeri* might be an example for the strategic nuptial gift allocation. Males exhibit condition dependent spermatophore investment, with heavier males transferring larger spermatophores. However, larger males allocate a lower proportion of their body mass to spermatophores than less heavy males [62]. Spermatophore investment is generally costly and therefore subject to limits. Previously mated males in various bushcricket species transfer smaller spermatophores than virgin males [63-67]. There is also a time constraint for males. Spermatophore size increases with male age at first mating [42,68] and the time since a previous mating [69-76]. Repeatedly mating males of the Australian *Requena verticalis* vary their mating investment over successive matings by reducing the total amount of spermatophore material [77], or by increasing their remating interval [70,78]. In two bushcricket species where investment in the spermatophore is relatively low, no change in spermatophore size occurred over consecutive matings [66,79]. In the relatively high spermatophore-investing *Ephippiger ephippiger*, males transferred similar sized spermatophores over the weeks, yet sperm number and nitrogen content was significantly reduced on a male’s fourth mating, indicating that male mating history influences a male’s investment over the mating season [68]. These laboratory-derived data were confirmed by measurements of spermatophore investment using field-collected males over the season. This approach allows investigation of the correlation between the time since last mating and the number of previous matings under a natural mating season. Only a few studies (all in the genus *Poecilimon*) have used field sampled bushcricket males. Whereas *P. affinis* males decrease their spermatophore weight over the season [72], there is only limited variation over a mating season in spermatophore size and spermatophore components including sperm number in two species of the *P. propinquus*-group [55]. Even if these closely related species differ in their relative investment, with *P. thessalicus* males investing more in relative and absolute spermatophore size than *P. v. minor* males, both species have surprisingly uniform spermatophore sizes over the season. Sperm number was not related to age for *P. thessalicus* even after correcting for ampulla weight [39]. There was a remarkable increase in ampulla weight without a corresponding increase in sperm number in *P. v. minor* at the end of the season; strengthening the idea that ampullae are not merely sperm containers, but are also sexual selected [55].

## Constraint between attraction calls and spermatophore provisioning

Bushcrickets are well known for their acoustic communication, which serves to attract mates and/or play a role in male-male competition. As such, songs are sexually selected characters [26,30]. Songs are energetically expensive to produce [29,80]. Insects in general increase their metabolic rates during singing [80] and insect species that sing over extended periods exhibit a higher resting metabolic rate [81]. Tettigoniids are no exception, as calling individuals have increased oxygen consumption, indicative of higher metabolism rates during song production [82,83]. The increase in metabolic rate scales with the amount of calling, suggesting a direct connection between calling rate and energy investment.

Two questions arise from the energetic costs associated with calling; (1) whether calling is restricted by energy reserves (or how easily energy needed for calling can be replenished). If energy does constrain calling then (2) is there a trade-off between calling and spermatophore production?

There is evidence that calling is limited by the energy uptake. Males from one tettigoniid species on a restricted diet drastically decreased the number of calls per day and produced calls of reduced energy [84]. Furthermore, males infected by condition-depleting maggots of acoustically orienting flies [85,86] were also constrained in song production [87,88]. Infected males called less and their singing was less attractive to females in the Greek bushcricket *P. mariannae*[89].

A trade-off between calling and spermatophore size was inferred from comparative studies that showed tettigoniid species producing calls with increased frequencies transferred comparatively smaller spermatophores [47]. This was also experimentally demonstrated in the tettigoniid species *Requena verticalis* when removal of the costs of song production led to increased spermatophore replenishment [69].

## Costs and benefits of nuptial feeding for bushcricket females

Females in sexual species generally need males to receive sperm. Since females can store sperm, one or a few matings should be enough to guarantee the fertilization of their ova [27]. Why then do females from many species have multiple mates [90], especially given the many costs associated with mating for females [1]? Costs include exposure to disease [91], predation [92,93], or physical harm from males [94]. Nevertheless, female accept matings with multiple males in a wide range of animals [95,96]. This behaviour has led to debates about the adaptive significance and evolutionary consequences of polyandry. One explanation might lie in benefits to females that arise from multiple matings [27,95-98]. Female insects can increase their fecundity through multiple matings, laying more eggs than singly mated females [99], especially in species that have nuptial feeding [97]. Where nuptial feeding occurs, multiple mating can increase egg and offspring production by as much as 35 to 85 percent [97]. This greater effect of polyandry on egg production in nuptial feeding species can be linked to increased nutrient resources transferred to females through multiple matings. Hatching success can also be increased by polyandry, but is independent of nuptial feeding. This may be due to the avoidance of sperm depletion [97].

In tettigoniids, spermatophylaces have been shown to influence female fecundity, through changes in egg number or egg mass. Studies in the Australian bushcricket *Requena verticalis* have shown that the number of spermatophylaces consumed increased the egg number and mass in food restricted [100,101] but not in well-fed females [102,103]. Similarly, in *Kawanaphila nartee* spermatophylax feeding only increased female fecundity if females were from a food-restricted habitat [65]. The reasons for increased egg production are unclear, but it seems reasonable to assume that nutrients from the nuptial gift allow females to produce more eggs.

A range of factors might stimulate or even manipulate females to lay more eggs. These included hormonal substances in the spermatophore [104-106], ejaculate quantity [99] and seminal fluid proteins [107]. Other factors that enhance female fecundity might be the greater numbers of sperm or the act of mating itself [97]. In the genus *Poecilimon,* the number of matings increased the egg-laying rate in one species [108], whereas the consumption of the spermatophylax in another species did not [109,110]. In an experiment with *Leptophyes punctatissima,* the increase in fecundity through multiple mating was independent of the nutritious aspect of spermatophylaces, as this increase was also found in females prevented from consuming any part of the spermatophylax [111]. In conclusion, spermatophores have been found to influence female fecundity positively in some species under some conditions, but there are equal numbers of studies reporting no effects (see Table 1). More subtle effects have also been reported. Spermatophore feeding in *Poecilimon veluchianus* increased the relative dry weight of eggs [109] and correlated positively with the lifespan of larvae in adverse conditions [110]. Similar results were found for *Requena verticalis*, where the size of consumed spermatophylaces increased egg weight and larger eggs showed a higher overwintering survival rate [101]. 

**Table 1:** Effect of mating number and nuptial feeding on female egg number or egg size in tettigoniids

			**Egg number**	**Egg mass**	
**Species**	**Character**	**Diet quality**	***Cohen's d***	***Cohen's d***	**Reference**
*Anabrus simplex*	Mating frequency	low, high	ns		[112]
*Conocephalus nigropleurum*	Mating number (double/single)	high	1.56		[113]
	Spermatophylax mass	high	ns		[113]
*Decticus verrucivorus*	Spermatophylax (yes/no)	low	ns	ns	[64]
*Kawanaphila nartee*	Spermatophylax (yes/no)	low	**4.14**	**2.10**	[65]
	Spermatophylax (yes/no)	low	**sig.**	**sig.**	[76]
*Leptophyes punctatissima*	Spermatophylax (yes/no)	high	-2.55 - 0.70	-0.39 - 0.33	[111]
	Mating number (double/single)	high	**3.29 – 9.35**	-0.39 - 0.33	[111]
*Leptophyes laticauda*	Spermatophylax (yes/no)	high	0.33 - 1.02	-1.60 - 1.20	[114]
*Poecilimon mariannae*	Mating number (double/single)	high	**0.83**		[108]
*Poecilimon veluchianus*	Spermatophylax (yes/no)	high	-1.70	2.66	[109]
*Requena verticalis*	Spermatophylax (yes/no)	high	-0.31	1.33	[102]
	Spermatophylax number (7/3/1/no)	low	**4.98-10.42**	1.77-4.85	[100]
	Spermatophylax number (3/1/no)	high	**sig.**	**sig.**	[63]
	Spermatophylax number (3/1/no)	low	**sig.**	**sig.**	[63]
	Spermatophylax mass (1.5/1/0.5)	high	ns	ns	[63]
	Spermatophylax (yes/no)	high	0.61	-1.83	[103]

I conducted a literature search for studies on female tettigoniid responses towards mating number, mating frequency, spermatophylax mass or the experimental provisioning of different numbers of spermatophylaces by using the *ISI web of science* and *Google scholar*. I also included articles mentioned in previous overviews [26,27,115] or a meta-analysis [97]. Data on nine tettigoniid species were found in 13 articles. I classified each study according to the experimental design used to compare females in different treatment groups. Females were tested in their responses towards: (1) mating frequencies with different males, (2) mating number, either single or double mated to two different males, (3) spermatophylax mass received during a single mating, or most commonly (4) the number of spermatophylaces consumed by single mated females, based on experimental studies that allowed or prevented female feeding on the spermatophylax after mating. For *Requena verticalis* experimenters varied the number of spermatophylaces between zero and seven. Because female responses might be higher in food-restricted conditions, I also specified the diet fed to females during the experiment as either high or low quality, based on the authors’ judgement. Figures in bold show significant differences between treatment groups according to the original references. For an independent measurement of effect sizes, I calculated the value of *Cohen's d*, using the means and standard deviations of two groups (treatment and control, [116]). *Cohen’s d* is positive, if the mean difference is in the predicted direction, with female fecundity increasing with mating number or spermatophylax feeding. Conventionally, a *Cohen’s d* of 0.2-0.3 is interpreted as a small, 0.5 as a medium and ≥ 0.8 as a large effect. Multiple data sets in the same study were presented as range. In cases where the reference does not allow calculation of *Cohen’s d* due to missing data, I indicated whether results were reported as significant (sig.) or non-significant (ns).

Apart from the influences on just female fecundity, interpretated as parental investment, the edible spermatophore is a high nutrient donation consumed by the females, and there is strong support that they provide direct benefits to females other than increasing offspring number or survival [117]. The spermatophylax of *Isophya kraussii* can supply the female her entire energy requirement for one to two days. As a female can mate every two to three days, she may obtain most of her food by mating [118]. Bushcricket spermatophores contain a reasonable amount of nitrogen, corresponding to a protein content of about 70 to more than 90% of the dry mass [119]. Carotenoids occur in spermatophores of *Ephippiger zelleri* and are known to increase survival and reproduction in some animals [120]. For many species, especially herbivores, nitrogen is a limited resource. Male-derived protein in the nuptial gift increased the nitrogen content of female muscle mass, indicating that spermatophore compounds are incorporated into the somatic tissue of females [121]. It has been shown that in the long-term females allocate spermatophore nutrients to either egg production or body synthesis [66,101,122,123]. By tracking stable carbon isotope ratios of female breath, Voigt et al. [124] found that the exhaled gas quickly converged on the ratio of the male donor, which were either enriched or depleted in ^13^C. This supports the idea that females can route nutrients to metabolism instead of egg production, according to their own and immediate needs without direct benefits for the male donor. Females of the obligate parthenogenetic *Poecilimon intermedius* mate with heterospecific males and subsequently feed on the nuptial gift, without changing the number or the hatching success of eggs [125]. These results in general support the view that nuptial gifts can also contribute to female homeostasis and thus promote female fitness. In this respect, nuptial gifts can be key determinants of female energy intake [126] and may have similar effects as host plant components in herbivorous insects [127]. Gifts clearly supply nutrients when females compete for them. Food-restricted females increase mating rate [65,112,128] or compete for gift-bearing males [129], showing a sex role reversal [65,130]. Given the benefits of nuptial feeding, females may try to choose a better spermatophore provider or increase the mating rate. However, the mating rate is somewhat limited, as each mating induces a female refractory period, mostly of several days. Therefore, the beneficial effects of spermatophylax feeding might be a strong force in shaping bushcricket mating systems, leading to strong female preferences of heavier, better-conditioned males as mates, which provide larger spermatophores [40-42].

In addition to the nutrients provided with the spermatophylax it is frequently observed that receptivity-suppressing compounds transferred in the ejaculate can have a negative impact on female lifespan [99,107,115]. In an experiment with *Requena verticalis*, females’ experienced reduced longevity when they were mated three times, compared to the longevity of single mated females [103]. The females used were mated to males but postmating prevented from feeding on the spermatophylax, receiving just the ejaculate and the sperm out of the ampulla. The authors argue that this reduction is not compensated by spermatophylax consumption. To give evidence for that, they mated a third group of females once and allowed to consume the spermatophylax, additionally feeding the females two additional spermatophylaces. These females lived as long as the once mated females prevented from spermatophylax consumption. Therefore, the authors [103] reject the hypothesis that potentially manipulative ejaculates can be compensated for by additional nutrients. However, double mated females receiving unmanipulated spermatophores, with both the ‘beneficial’ spermatophylax and the ‘detrimental’ ejaculates, showed a remarkable increased lifespan in another tettigoniid species, compared to single mated females [113]. Obviously, we can still learn a lot from the effects of the different parts of the spermatophore on multiple female responses, and there is a need for studies testing these effects directly.

## Protandry and sperm competition

Life history models typically assume that there are benefits of larger adult size, as large adults show greater competitive ability as well as increased fecundity. However, it takes time to grow to a large size and, assuming a constant mortality rate, a long juvenile period decreases the survival rate, leading to a trade-off between age and size at maturity [131]. There is widespread evidence for growth strategies to be influenced mainly by food availability, time constraints and the mating system. Restrictions in food intake limit body size and can be caused by competition between members of the same food guild or due to predation risks, lowering activity patterns connected with foraging. The duration of development is largely constrained by seasonality, where a life stage has to be reached by a particular time [131,132]. Optimal size and age at maturity differ between species but also within species, as the size of males and females is influenced by different selection mechanisms. It is generally agreed that large female size is primarily fecundity selected and large male size primarily sexually selected [133]. In females, the number or quality of offspring increases with body size [134,135]. Therefore, females are selected to maximize their body size, despite the costs of increased time used for foraging and delayed maturation. Larger males can gain in reproductive success both through male–male competition and through female choice [1]. This differential selection on females and males will cause differences in the direction and degree of sexual size dimorphism [136].

In a large number of insects, males generally moult earlier than females into adulthood, a phenomenon known as protandry [137]. It has been proposed that protandry may be best explained as a result of sexual selection. Early reproductive age might contribute to the competition ability of mates and it is hypothesized that protandry is an adaptive strategy to increase the males reproductive success [138-140]. Males may be under stabilizing selection for a degree of protandry that maximizes the number of females mated or, in polyandrous mating systems, the number of virgin females mated [141]. The pattern of sperm competition to be biased towards the first or the last male might select for the duration of the time shift between male and female maturation. Where there is first male sperm precedence, selection for early male maturation may be strong despite costs associated with being smaller. Thus, a relatively short time window for reproduction or a decline in the reproductive value of females over the season may result in extended protandry. The reverse must be true for last male sperm precedence, where enhanced size dependent male-male competition for females [1], may decrease the value of early maturation and therefore restrict protandry [137,141].

**Figure 3:**
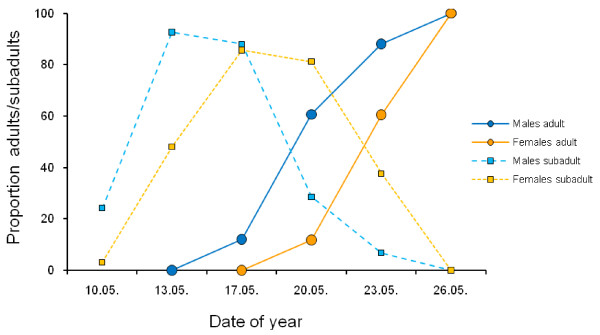
**Protandry in the Greek bushcricket*P. mariannae*at Vrissia.** Males moulted into adulthood in mean 3 days earlier than females (calculated at 50 percent proportion of adults). Sample sizes for males (n = 33, 55, 25, 28, 56, 57) and females (n = 32, 50, 42, 85, 61, 63).

In tettigoniids, protandry is seldom acknowledged in the literature about mating systems [142,143]. The extent of the protandry, however, is extremely variable, ranging from a few days in the genus *Poecilimon* (Figure3) to more than a month in the Australian *Requena verticalis*[143]. This variability in the extent of protandry correlates with life-history parameters (Table 2). The extent of the protandry correlates across the species with the sperm competition pattern; the genus *Poecilimon* has a relatively high rate of last male sperm advantage [144,145], with males moulting one to three days earlier into adulthood than females. In contrast, *R. verticalis* males appear more than a month before their females [143]. This pattern might be attributed to the type of sperm competition in *R. verticalis*, with an almost complete first male sperm advantage, proposed to result from a complete filling of the female spermatheca with the sperm of the first male [146]. As such, the results are in line with the hypothesis generated from the butterfly data [137,141] or proposed for spiders [147]. 

**Table 2:** Protandry across bushcricket species

**Species**	**Length of**	**Ref.**	**Protandry**	**Sample size**	**Origin of individuals**	**Ref.**	**Sperm**	**Ref.**
	**adult season**		**[days]**	**(males, females)**			**competition**	
*Decticus verrucivorus*	3-4 months	[64]	≤ 10 days	115, 106	ex nymphs, lab reared	[142]	sperm mixing	[148]
*Poecilimon aegaeus*	4 weeks	*	0.6 days	32, 21	ex nymphs, lab reared	*	last male precedence	[144,145]
*Poecilimon ampliatus*	4-6 weeks	*	1.3 days	150, 63	ex nymphs, lab reared	*	last male precedence	[144,145]
*Poecilimon mariannae*	4 weeks	[89]	3 days	25-57, 32-85	field samples	**	last male precedence	[144,145]
*Requena verticalis*	10 weeks	[143]	35 days	14-21, 2-14	field samples	[143]	first male precedence	[146]

The difference, measured in days, between the mean appearance (50%) of mature males and females from lab reared or field collected specimens.

*Lehmann unpublished, data on file, **this article Figure3.

Selection acting on males in relation to sperm competition might be a driving force for protandry in bushcrickets. However, this conclusion is drawn from a correlation between sperm precedence pattern and the extent of protandry based on a restricted number of species (Table 2). Further studies are required to support or refute this hypothesis. In particular, experimental tests of the growth pattern of bushcrickets would be valuable to identify critical influencing factors.

## Mate density and sampling costs

Environmental factors influence sexual selection and the evolution of mating systems [3,149]. This section concentrates on the number and density of potential mates, as not only the quality of a sex partner but its accessibility might be important for understanding mating decisions. With an increasing mate density, more possible mates can be screened, saving time and energy, and reducing predation risks associated with searching for mates [140]. Reduced mate densities are likely to increase the costs of mate choice [140]. The spatial distribution of potential mates makes it necessary to assess mates sequentially rather than simultaneously. Nonetheless, there is evidence that individuals of a large number of animal species actively choose between mating partners [150-153]. As mate choice can be costly in terms of time and energy expenditures [154,155] or increased susceptibility to predation [156-161], it might be advantageous to accept mates above some critical quality threshold. There is evidence that the decision to accept a given mate is plastic and constantly adjusted to the expected return from continued searching [162-164]. With low search costs, such an “adjustable threshold” will result in the acceptance of a high-quality mating partner. If costs increase, individuals will accept a partner of lower quality to minimise those search costs.

In tettigoniids, population density influences the predation risk during mate searching as mortality increases with travel distance in non-flying species [165,166]. The encounter rate of potential mates can be used as a proxy for population density in laboratory experiments. Indeed, male mating behaviour changed with previous female encounter rate in two Australian bushcricket species, *Kawanaphila nartee*[167] and *Requena verticalis*[168]. In field enclosures with differing mate densities, female *Xederra charactus* bushcrickets adjusted the tactic of sequential mate sampling in response to mate density [169]. In populations of high density, females approached more males sequentially, but at lower density, they were less choosy. Individuals might also reduce choosiness as the mating season advances. The more individuals that have mated and therefore dropped out from the pool of available mates the lower is the likelihood in finding a mate of better quality during extended sampling [170]. *Xederra charactus* females were less likely to reject mates later in the night when population density was low. This behavioural change is consistent with a flexible adaptation to the time constraints of a finite nightly mating period. As a result, high density allows females to choose from a larger pool of males, leading to a mating advantage for males having a higher mass of the spermatophore secreting glands. As this glandular a responsible for the production of the spermatophore, females benefit by receiving a larger nuptial gift at mating [169]. These results are in line with tactical models of search behaviour, which predict an adjustment of female behaviour to the number of potential mates and the length of the mating period [170]. A less explored field is the integration of sampling costs into the mating game in general [9]. A few field studies using tettigoniids demonstrate the value of this approach [113,169].

## Conclusions

This review highlights what is known about costs and benefits of traits involved in mate choice in bushcrickets. Such studies are important because they allow us to identify the traits, which are subject to selection, and to interpret comparative and experimental evolutionary studies. In this review, I have identified four areas of the mating economy that would benefit from further research. Firstly, the spermatophore components have different functions and their scaling with male body size or male condition differs. Further investigation is required to identify and tease apart selective pressures associated with the spermatophylax and ampulla and the number of sperm. Given inter-population variability in body size and sexual size dimorphism further research into population variation in reproductive strategies, especially the male spermatophore investment patterns, would be useful. Thirdly, protandry is a poorly studied topic in bushcrickets, it remains to be confirmed whether protandry and the sperm utilizing patterns in tettigoniids matches theory and results from other taxonomic groups. Finally, this review also served to underscore the importance of environmental effects and individual condition on mate choice. We still need careful experiments to identify which factors are important in particular species by connecting the mating strategies of the sexes with their life history.

## Competing interests

The author declares that she has no competing interests.

## Authors’ contributions

GL wrote the entire manuscript.
